# China’s private institutions for the education of health professionals: a time-series analysis from 1998 to 2012

**DOI:** 10.1186/s12960-018-0308-6

**Published:** 2018-08-22

**Authors:** Jianlin Hou, Zhifeng Wang, Youhui Luo, Joseph C. Kolars, Qingyue Meng

**Affiliations:** 1 Institute of Medical Education & National Center for Health Professions Education Development, Peking University, Beijing, China; 2 School of Public Health, Peking University, Beijing, China; 3 Office of Education, Peking University Health Science Center, Beijing, China; 4 University of Michigan Medical School, Ann Arbor, MI United States of America; 5 China Center for Health Development Studies, Peking University, No. 38 Xueyuan Road, Beijing, 100083 China

**Keywords:** Private education institution, Education of health professionals, Scale of education, Structure of education, Educational resources, China

## Abstract

**Background:**

Public institutions have been the major provider of education for health professionals in China for most of the twentieth century. In the 1990s, the Chinese government began to encourage the establishment of private education institutions, which have been steadily increasing in numbers over the past decade. However, there is a lack of authoritative data on these institutions and little has been published in international journals on the current status of private education of health professionals in China. In light of this knowledge gap, we performed a quantitative analysis of private institutions in China that offer higher education of health professionals.

**Methods:**

Using previously unreleased national data provided by the Ministry of Education of China, we conducted time-series and descriptive analyses to study the scale, structure and educational resources from 1998 to 2012 of private institutions for health professional education.

**Results:**

The number of private institutions that educate health professionals increased from two in 1999 to 123 in 2012. Private institutions displayed an average annual growth rate of 44.2% for enrolment, 59.0% for the number of students and 53.3% for the number of graduates. In 2012, nursing, clinical medicine and traditional Chinese medicine had the most students (37.2%, 32.8% and 8.9% respectively), representing 78.9% of all students in these institutions. Ninety-seven private institutions located in the more economically advantaged eastern and central China and only 26 ones were in the less economically advantaged western China, respectively turning out 85.2% and 14.8% of health professional graduates. There were less educational resources, such as the number of faculty members, physical space and assets, at private institutions than at public institutions.

**Conclusions:**

Private institutions for the education of health professionals have emerged quickly in China, contributing to the demand for health professionals that exceeds what public institutions are able to offer. At the same time, the imbalance of geographical distribution and poor educational resources of private institutions are of concern. It may be of utmost importance to enhance administration and supervision to better regulate private institutions and their development plans. Future studies may be needed to better examine the effects of private institutions on the production and allocation of health workers.

## Background

The high cost of educating health professionals is a major issue for public institutions that provide higher education. By allowing private capital into this sector, the government’s financial burden is reduced and the number of health profession graduates can increase without further public investment. Globally, the contribution of private institutions to the education of health professionals varies among countries and regions [1]. In many countries, non-profit and for-profit private education institutions have become an important component for health-professional education and often account for the greatest area of growth. For example, most of the new medical schools built in India since the 1970s were private institutions [1]. And the first private medical school in Sub-Saharan Africa was founded in 1990 where private medical schools now account for 26% of all medical schools [2]. In other countries such as the United States of America and Japan, private medical schools account for a sizable portion of the accredited medical schools that offer terminal degrees [3, 4]. Also, in the United Kingdom, the first private medical school began admitting students in 2015, followed by an intention to start another private medical school by a public university [5].

In China, private medical schools were first established by missionaries from western countries in the mid-nineteen century [6, 7]. After the founding of the People’s Republic of China in 1949, the government took over control of all public and private education institutions. Subsequently, private institutions were closed or transformed into public institutions which exclusively oversaw the education of health professionals for nearly half a century. In October 1997, China’s State Council issued new *Regulations on the Running of Education Institutions with Social Resources* which encouraged the further development of private education institutions while maintaining strict control over the regulatory aspects of higher education [8]. After 2003, with the implementation of *Private Education Promotion Law of the People’s Republic of China*, followed by *The Regulation on the Implementation of the Private Education Promotion Law of the People’s Republic of China*, individuals and non-governmental sectors were encouraged to invest in education. With the removal of restrictive regulations, the number of private institutions offering health professional education increased rapidly [9]. According to laws and regulations in China, private education belongs to the public and their welfare and, therefore, should not be for profit. However, those providing the financial capital were allowed to “obtain a reasonable amount of requital from the surplus” after the “cost of a private school is deducted…and the sum of money for other necessary expenses is drawn in accordance with the relevant regulations of the State” [10].

At present, private institutions in China that offer higher education for future health professionals are established through two different approaches which are typically complex with regard to how they were established, ownership and models of operation [9]. The first approach consists of entities such as individuals, social groups, or a school offering a major of applied techniques (e.g. rehabilitation, acupuncture) at the level of higher vocational training leading to the equivalent in the West of a junior college degree (i.e. *Da Zhuan* in China). The other approach consists of a public university or college establishing a private arm of the institution, often referred to as an “independent school,” that most typically offers an undergraduate education programme leading to a bachelor degree [9]. Public universities following this second approach commonly partner with other parties, such as local governments, enterprises, individuals and social groups to establish an independent school [11]. The Ministry of Education (MOE) of China issued *Measures for the Establishment and Administration of Independent Schools* in 2008, which defines independent schools as “schools engaging in undergraduate education which are jointly established by regular universities and colleges engaging in undergraduate education or above with non-government sectors or individuals with funding from sources other than national finance” [12].

Historically, government funding in China supported public education. On rare occasions, private institutions draw government funding which is usually extremely small. Due to the lack of preferential tax policies for donors, donation revenues for private education institutions are limited. Only a few private institutions offering health profession education have relatively sufficient funding. These institutions employ experienced administrative and teaching staff from public universities and are therefore more familiar with modern management systems and problem-solving abilities compared to other private institutions. These institutions with sufficient funding and staff have demonstrated their contributions to the education of health professionals in China [13–15]. However, due to the lack of funding as well as a strong profit orientation, investments in human and material resources by many other private institutions are insufficient, resulting in deficiencies across their education systems.

First, most private institutions generally offer poor remuneration making it difficult to attract and retain quality faculty members. Many of these are full-time employees of public institutions who only teach at private institutions on a part-time basis. With a high turnover rate and a large percentage of part-time faculty, private institutions are unable to compete with public institutions in terms of academic research and teaching quality [16, 17]. Private institutions are also challenged to define their discipline and curriculum while staying up-to-date with education reform in the country [16–18]. Second, in addition to the lack of teaching materials, many laboratories at private institutions are in poor conditions and cannot accommodate the practical learning needs of their students. Some private institutions also lack proper infrastructure and buildings, such as classrooms and dormitories [19–21]. Third, clinical teaching bases (e.g. affiliated or teaching hospitals) are often not accessible to students who attend private institutions. Due to high construction cost, many private medical schools do not have their own affiliated or teaching hospitals, which poses substantial inconveniences for clinical teaching and training [19, 21–24]. All of these issues contribute to the failure of private institutions’ ability to deliver rigorous, high-quality education, perpetuating negative stereotypes towards private education of health professionals, resulting in local government unwilling to provide support [25]. In addition, faculty from private institutions is not held in high regard when competing with faculty from public institutions for positions [20]. There is also discrimination against health professional graduates from private institutions, which is reflected in hiring policies or standards established by some local governments in the recruitment of public servants [26–28].

Even though private institutions that train health professionals have been developing for over a decade in China, little has been documented in the literature. Domestic studies have been primarily qualitative descriptions or with students as study objects. To the best of our knowledge, there is a lack of authoritative statistics and analyses on the nationwide development of private institutions for the education of health professionals in China, particularly in international peer-reviewed journals. In the present study, we aimed to use national data to conduct a quantitative analysis of the scale, structure and educational resources of the private institutions offering higher education of health professionals. Furthermore, we sought to compare private and public institutions in terms of their changes in higher education of health professionals, educational resources and relative contribution to the production of health workers. In the following sections of the paper, we described the data and analysis method used in the study, presented results of data analysis, discussed policy implications of the findings and summarized major conclusions.

## Methods

The data used in this study are previously unreleased national routine data provided by the MOE of China, including the (1) number of health professional students by school and major degree and (2) statistics on faculty and staff size, assets and physical space by school. Descriptive analysis was performed from datasets generated from yearly statistical forms between 1998 and 2012 that each higher education institution submits to the MOE and local education authorities. Select aggregate results of these forms are accessible to the public, but no national statistics on private institutions for the education of health professionals have been released. The datasets that we obtained from the MOE consist of all institutions offering higher education of health professionals. The total number of students and faculties in the datasets equal those in the aggregate results that are assessable to the public. The complete and accurate MOE datasets enable us to define the quantitative characteristics of these institutions and their changes in the country.

The focus of our analysis within the MOE datasets is private regular institutions offering higher education of health professionals. Regular is defined as educational institutions with health science majors including all of the following 11 first-level disciplines in the education of health professionals: basic medicine, clinical medicine, pharmacy, public health and preventative medicine, nursing, traditional Chinese medicine (TCM), stomatology, medical techniques, Chinese pharmacy, forensic medicine and integrated Chinese and western medicine. These institutions provide programmes that lead to a junior college (i.e. *Da Zhuan)*, bachelor, master, or doctorate degree. The length of the education programmes varies from 3 to 8 years.

China’s higher educational institutions (HEIs) consist of regular HEIs and HEIs for adults [29]. Typically, the former admits high school graduates as full-time students through the national college entrance examination, while the latter provides adults with higher education on a part-time basis through distance education or some on-campus courses. There was only one private HEI for adults in 2015, according to statistics from the Ministry of Education of China. Only private regular HEIs are included in our analysis because they are dominant in and most representative of China’s higher education of health professionals.

## Results

### Overall scale of education and its changes

As noted in Table 1, since the emergence of two private colleges in 1999, the total number has increased to 123 in 2012, accounting for 20.8% of all institutions offering higher education of health professionals. Total enrolment in these private institutions rose from 745 in 1999 to 86 501 in 2012. The total number of health professional students surged to 310 386 compared with 745 in 1999, while the number of graduates climbed up to 70 868 in contrast to 991 in 2002. During the time span of 1999 to 2012, private institutions were seeing an average annual growth rate of 44.2% for enrolment, 59.0% for number of students and 53.3% for number of graduates, significantly higher than public ones (11.9%, 13.4% and 15.5% respectively). As for percentages in the education of health professionals, private institutions were also seeing a yearly increase, with percentage of enrolment, students and graduates respectively reaching 14.7%, 14.8% and 14.0% in 2012.

**Table 1:** Number and percentage of private and public education in China’s higher education system of health professionals: 1998–2012

	Institution		Total number of enrolments		Total number of students		Total number of graduates	
Year	Private	Public	Private	Public	Private	Public	Private	Public
1998	0 (0.0)	189 (100.0)	0(0.0)	82 138 (100.0)	0 (0.0)	301 866 (100.0)	0 (0.0)	65 916 (100.0)
1999	2 (1.0)	207 (99.0)	745 (0.6)	116 117 (99.4)	745 (0.2)	350 078 (99.8)	0(0.0)	66 763 (100.0)
2000	3 (1.1)	261 (98.9)	941 (0.6)	160 241 (99.4)	1 505 (0.3)	448 939 (99.7)	0(0.0)	65 682 (100.0)
2001	4 (1.4)	282 (98.6)	3 153 (1.7)	186 706 (98.3)	5 263 (0.9)	560 883 (99.1)	0(0.0)	69 259 (100.0)
2002	20 (6.3)	295 (93.7)	7 384 (3.3)	214 896 (96.7)	13 854 (2.0)	680 497 (98.0)	991 (1.1)	85 835 (98.9)
2003	60 (15.4)	330 (84.6)	22 191 (8.0)	254 800 (92.0)	43 270 (5.0)	820 645 (95.0)	1 576 (1.3)	119 340 (98.7)
2004	86 (19.0)	366 (81.0)	34 911 (10.9)	285 373 (89.1)	78 208 (7.6)	950 845 (92.4)	4 969 (3.0)	162 105 (97.0)
2005	98 (20.9)	372 (79.1)	50 010 (13.7)	315 026 (86.3)	118 835 (9.9)	1 081 519 (90.1)	7 615 (3.5)	208 562 (96.5)
2006	103 (21.1)	384 (78.9)	52 922 (12.9)	357 326 (87.1)	152 484 (11.3)	1 196 932 (88.7)	15 980 (5.9)	254 867 (94.1)
2007	112 (21.7)	405 (78.3)	59 404 (14.9)	339 281 (85.1)	193 810 (13.1)	1 285 656 (87.9)	27 460 (8.5)	295 599 (91.5)
2008	110 (21.4)	405 (78.6)	66 052 (15.3)	365 660 (84.7)	222 145 (13.7)	1 399 351 (86.3)	40 447 (10.2)	356 092 (89.8)
2009	105 (20.2)	416 (79.8)	78 501 (16.1)	409 083 (84.9)	255 331 (14.6)	1 493 511 (85.4)	49 714 (12.0)	364 569 (88.0)
2010	110 (20.4)	429 (79.6)	80 062 (15.3)	443 219 (84.7)	274 650 (14.6)	1 606 514 (85.4)	60 344 (12.9)	407 439 (87.1)
2011	119 (20.9)	451 (79.1)	84 309 (14.4)	499 334 (85.6)	294 534 (14.9)	1 677 040 (85.1)	65 877 (13.5)	420 761 (86.5)
2012	123 (20.8)	467 (79.2)	86 501 (14.7)	500 036 (85.3)	310 386 (14.8)	1 793 241 (85.2)	70 868 (14.0)	436 790 (86.0)

Note: percentages in parenthesis

**Fig. 1:**
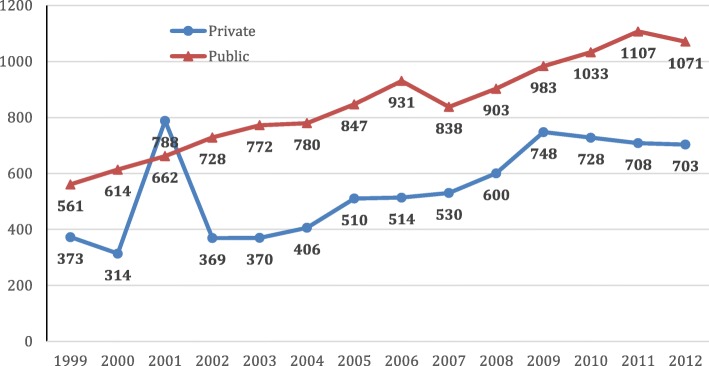
Average student enrolment in private and public institutions in China’s higher education of health professionals: 1999–2012

From 1999 to 2012, the average enrolment of private institutions increased from 373 to 703. In the same period, the average enrolment of public institutions rose from 561 to 1 071. Except for 2001, the average enrolment of private institutions was less than that of public ones (Fig. 1).

### Structure of education

Independent schools, namely private arms of public universities or colleges, grew faster than other kinds of private institutions. Among 123 private institutions in 2012, 57 (46.3%) were independent schools, 37 (30.1%) were higher vocational schools and 24 (19.5%) were colleges. Undergraduate education was available in 76 (61.8%) private institutions while 78 (63.4%) ones offered junior college education. None of the 123 institutions has master or doctoral programmes, indicating that private education of health professionals is limited to junior college and undergraduate level.

Of the 123 institutions, 61 were in eastern China, 36 in central China and 26 in western China, respectively turning out 53.7%, 31.5% and 14.8% of health professional graduates. Eastern China has the most private education institutions and graduates, central China less and western China least. Such geographical distribution pattern is the same with public education institutions (Table 2).

**Table 2:** Geographical distribution of private and public education institutions and their health professional graduates in China: 2012

	GDP per capita (yuan)	Total population (million)	Institution		Graduate	
			Private	Public	Private	Public
Eastern	57 429	558.5 (41.4)	61 (49.6)	200 (42.8)	38 040 (53.7)	183 651 (42.0)
Central	33 382	425.1 (31.5)	36 (29.3)	159 (34.0)	22 334 (31.5)	159 443 (36.5)
Western	31 269	364.3 (27.0)	26 (21.1)	108 (23.1)	10 494 (14.8)	93 696 (21.5)
Total	42 580	1 347.9 (100.0)	123 (100.0)	467 (100.0)	70 868 (100.0)	436 790 (100.0)

Note: Data source for GDP per capita and total population: China Statistical Yearbook 2013; percentages in parenthesis

Private institutions do not put as much emphasis on research programmes or on preparation for academic careers as much as public institutions. Instead, they focus on the education of health professionals that are popular and have a promising career prospection, or offer programmes with a low cost or requirement of educational resources, such as nursing, TCM and the education of practical clinical professionals for rural areas and regions of lower administrative level (usually referred to as “grass-root level”) [22, 25, 30, 31].

In 2012, nursing, clinical medicine and TCM have the most students (37.2%, 32.8% and 8.9% respectively), adding up to 78.9% of all students in private institutions. In terms of percentage of private health professional students nationwide by major, it is stomatology (22.6%), integrated Chinese and western medicine (20.4%) and TCM (17.9%) that prevail (Table 3).

**Table 3:** Health professional students of private education institutions by major in 2012

Major	*N*	Percent	% of students in the whole country
Nursing	115 550	37.2	17.5
Clinical medicine	101 863	32.8	13.9
TCM	27 593	8.9	17.9
Pharmacy	18 329	5.9	10.2
Stomatology	16 393	5.3	22.6
Medical techniques	15 536	5.0	11.9
Integrated Chinese and western medicine	9 326	3.0	20.4
Chinese pharmacy	4 736	1.5	7.9
Public health and preventative medicine	920	0.3	1.8
Forensic medicine	140	0.0	2.6
Basic medicine	0	0.0	0.0
Total	310 386	100.0	14.8

**Fig. 2:**
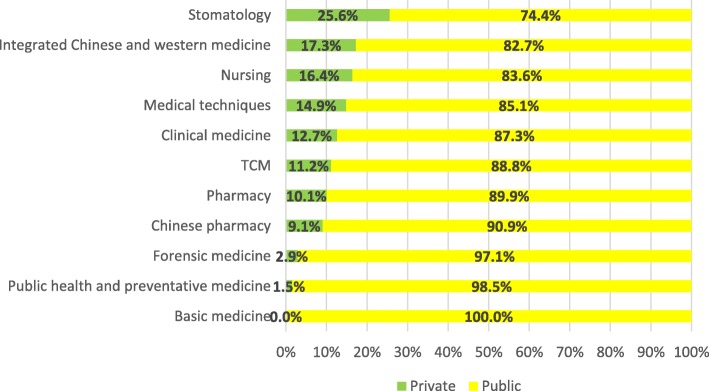
Percentage of health professional graduate of private and public education institutions by major in 2012

**Fig. 3:**
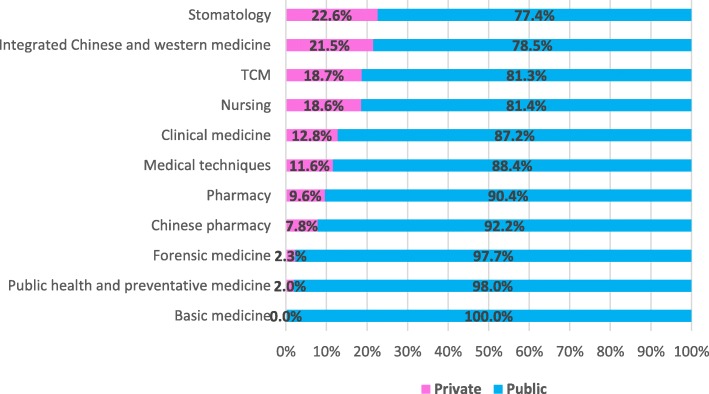
Percentage of health professional enrolment of private and public education institutions by major in 2012

As for health professional graduates nationwide by major, private institutions were seeing a relatively large proportion in stomatology (25.6%), integrated Chinese and western medicine (17.3%) and nursing (16.4%). By contrast, a majority of health professional graduates were turned out by public institutions in all of the 11 majors with the biggest three percentages found in basic medicine (100%), public health and preventive medicine (98.5%) and forensic medicine (97.1%) (Fig. 2). Such discipline distribution is similar to health professional enrolment (Fig. 3).

### Educational resources

Education resources largely decreased in private institutions from 1999 to 2012, except multi-media classroom seats per hundred students. Specifically, student-faculty ratio increased more than fourfold, from 5.2:1 to 23.9:1. Gross value of fixed assets per student fell from 439 000 yuan to 40 000 yuan (i.e. 67 518 to 6 152 in 2016 US dollars). Value of teaching and research devices and equipment per student decreased from 19 000 yuan to 7 000 yuan (i.e. 2 922 to 1 077 in 2016 US dollars) (Table 4).

**Table 4:** Educational resources of private institutions for the education of health professionals: 1999–2012

Year	Student-faculty ratio	Teaching and administrative buildings (m^2^ per student)	Campus area (m^2^ per student)	Dormitory area (m^2^ per student)	Classroom area (m^2^ per student)	Gross value of fixed assets (10 000 yuan per student)	Library books (per student)	Teaching computers (per 100 students)	Seats in multi-media classrooms (per 100 students)	Value of teaching and research equipment (10 000 yuan per student)
1999	5.2	35.1	1 061.9	38.3	13.4	43.9	135.6	–	–	1.9
2000	7.2	28.2	442.9	24.1	13.1	6.5	118.3	–	–	1.0
2001	15.4	61.2	256.0	16.8	39.3	10.8	193.9	–	–	0.6
2002	13.3	19.7	105.6	10.5	10.1	4.5	58.7	20.1	54.9	0.4
2003	14.9	17.9	97.8	11.2	8.7	4.3	45.3	20.6	62.5	0.6
2004	17.4	17.6	79.8	9.8	9.0	3.8	53.2	17.9	67.2	0.6
2005	20.4	13.8	65.8	8.6	6.0	3.7	53.6	13.1	76.9	0.5
2006	22.2	14.6	63.5	8.8	6.4	3.6	55.4	14.1	71.7	0.5
2007	24.0	8.1	41.8	5.1	4.0	3.1	51.6	13.6	67.5	0.4
2008	24.6	5.2	26.2	3.1	2.6	3.3	60.7	14.6	72.7	0.5
2009	25.1	6.8	30.0	3.4	3.2	3.2	63.6	14.4	72.1	0.6
2010	26.0	6.6	29.9	3.0	3.3	3.3	66.6	15.9	77.4	0.6
2011	24.7	12.4	59.9	10.7	4.5	5.2	74.0	28.9	91.0	1.6
2012	23.9	8.4	40.4	6.2	4.2	4.0	75.1	18.5	94.2	0.7

As noted in Table 5, private institutions do not compare favourably with public ones, in nearly all primary educational resource indicators.

**Table 5:** Comparison of educational resources between private and public institutions for the higher education of health professionals

	2012			National standard^1^	Average rate of change (%): 1998**–**2012		
	Private	Public (central government)	Public (others)		Private	Public (central government)	Public (others)
Student-faculty ratio	23.9	12.8	20.3	16.0	11.5	7.1	5.5
Campus area (m^2^ per student)	40.4	81.5	70.0	59.0	− 20.9	− 1.6	− 0.3
Teaching and administrative buildings (m^2^ per student)	8.4	16.0	14.6	16.0	− 9.3	1.2	0.8
Classroom area (m^2^ per student)	4.2	3.2	5.3	–	− 8.3	2.2	2.3
Dormitory area (m^2^ per student)	6.2	14.6	11.2	6.5	− 12.3	11.7	8.3
Gross value of fixed assets (10 000 yuan per student)	4.0	8.1	4.4	–	− 16.0	9.6	9.2
Library books (volume per student)	75.1	80.0	67.9	60/80	− 4.5	− 2.4	− 1.9
Teaching computers (per 100 students)^2^	18.5	34.7	25.3	8.0/10.0	− 0.8	6.5	11.3
Seats in multi-media classrooms (per 100 students)^3^	94.2	95.6	91.5	7.0	7.2	18.2	21.1
Value of teaching and research devices and equipment (10 000 yuan per student)	0.7	3.4	1.2	0.4/0.5	− 7.4	18.5	10.8

^1^Ministry of Education of China, 《Qualification indicator of educational resources for universities and colleges》, 2004

^2,3^Average rate of change for year 2002 to 2012 were calculated

## Discussion

Since 1999, private institutions have emerged and expanded to about one fifth of all institutions that educate health professionals in China. As of 2012, they accounted for nearly one seventh of the country’s health professional graduates. These private institutions have made significant contributions to the education of health professionals in shortage areas. We also show the impact of private institutions on specific cadre. For example, dental, TCM and nursing students of private institutions respectively accounted for 22.6%, 18.5% and 17.5% of students in the whole country in 2012.

As observed in India and many other countries, government investment on public education institutions alone could not keep up with the increasing demand for the education of health professionals [1–4]. With public education institutions having a history of more than 60 years in China, more attention may need to be placed on supporting and standardizing private institutions if they are expected to play a more meaningful role in producing health professionals for China. Funding is the vital material foundation for the development of any education. Like private medical schools in Africa and other places [2], the financial viability of private institutions in China mainly relies on investment of the operators and revenues from tuition fees. Although the primary data we could access does not report on the tuition or entrance college examination scores, the literature suggests that private institutions typically have higher tuition rates and lower entrance examination scores relative to their public institution counterparts [26, 32]. Therefore, some students choose to study in private institutions when they do not gain admittance to public ones. It was reported that private institutions in many provinces increased their tuition by about 30% or 50% in the past few years because they were allowed to set criteria of tuition fees by themselves, discouraging students from choosing to study in these institutions [33–35]. An additional financial burden that is often hidden relates to the fact that many students extended their high school period for a year or more in order to better prepare the college entrance examination and gain admittance to a public institution [33–35]. It will be essential for private institutions to set up a multi-channel and effective fund-raising system. In addition, the government should issue and implement policies of financial support for private institutions in accordance to the rule of health professional education, such as preferential tax policies for donors, supportive policies for application of research funding and tax reduction or exempt.

Worldwide, the distribution of medical schools has been closely associated with economic performance, which means that more developed regions tend to have more medical schools [1]. Such distribution patterns are also applicable in China. As shown in Table 2, there were more private education institutions and health professional graduates from the more economically advantaged eastern and central regions of China. We found 97 private institutions located in these two regions and only 26 ones were in the less economically advantaged western China, respectively turning out 85.2% and 14.8% of health professional graduates. Parameters of health in western China are lower as are the allocation of human resources for health. For example, there are only 1.7 registered nurses per 1 000 population in western China, compared with 2.0 in eastern China and 1.8 in central China.

Western China would benefit from efforts to strengthen the local education of health professionals. In 2012, the number of health-professional education institutions per ten million people in western China was 3.7, compared with the eastern (4.8) and central (4.6) regions of the country. Health professional graduates per hundred thousand people in the west were less 28.6, compared with the eastern (41.1) and central (43.1) regions [36]. If China does not increase investments for public institutions in the western areas, private institutions may attempt to fill this gap.

Educational resources are of utmost importance to guaranteeing the supply and quality of education [37, 38]. Education of health professionals often means a need for better educational resources. As the scale of education goes up, education institutions for health professionals in China are seeing a decline in educational resources, with per student educational resources reduced or hardly increased [39–41], in wake of which that of private institutions are least assuring. Therefore, in sacrificing needed educational resources for profits, private institutions for the education of health professionals might go farther than public ones. The assurance of educational resources and education quality calls for governmental guidance and supervision. Government should make clear basic requirements for establishment and be strict with the examination and approval of private education institutions, thus laying the foundation for education quality. At the same time, different regions may need to be treated differentially. For less developed regions, requirements for establishment may be compromised for the actual circumstances to a reasonable extent.

Although private institutions play an increasing role in the education of health professionals in China, their strong motives for profits, variability in quality and uneven distribution across the country raise concerns. To better regulate private institutions and their development plans, it may be of utmost importance to enhance administration and supervision, especially through enhancing accreditation of institutions and programmes to ensure that they meet a set of established standards [42, 43]. In the past few years, accreditation entities and mechanism have been established for several undergraduate programmes (e.g. medicine and nursing) in China. To date, only a few medical programmes of private institutions have been accredited though the MOE plans to complete first-round accreditation of all medical programmes by 2020 are in place.

This current descriptive analysis of private health profession schools in China leads to other important questions that should be pursued; the most important of which are the percentage of these graduates actually enter the health workforce and how they perform as health care professionals. Future studies should also examine where these graduates are practicing and their ability to secure position they desire. Qualitative studies including interviews with students, graduates, faculty and administrators of private institutions as well as with government officials are necessary to better interpret the quantitative data presented in this paper.

Although we present data from a unique dataset from the MOE in China, several limitations are worth noting. First, the datasets contain only the number of full-time faculty and not those who teach on a part-time basis. Therefore, the student-teacher ratio calculated in the study may be higher if this part-time faculty could be considered. Second, private institutions, especially in their early stages of development, may lack the staff and expertise regarding data, thereby limiting data quality. As it is difficult to address such issues when using national routine data, a sampling study and field survey of private institutions may be needed to further refine findings in the study.

Finally, some most recent developments regarding private institutions are also worth mentioning. With the investment of foreign capital and involvement from top international universities, some China-based overseas educational institutions such as Duke Kunshan University have recently emerged but not included in the data set. These models involve collaborations between public institutions in China and foreign schools that typically receive funding (e.g. land, infrastructure) from local government. These institutions operate privately and have the potential to contribute significantly to health profession education in China as they are capable of offering world-class quality of education. In this sense, they differ from the “traditional” private institutions we analysed in the paper.

## Conclusions

Private institutions for the education of health professionals have emerged quickly in China, contributing to the demand for health professionals that exceeds what public institutions are able to offer. At the same time, the imbalance of geographical distribution and poor educational resources of private institutions are of concern. It may be of utmost importance to enhance administration and supervision to better regulate private institutions and their development plans. Future studies may be needed to better examine the effects of private institutions on the production and allocation of health workers.

## Abbreviations

GDP: Gross domestic product
HEI: Higher educational institutions
MOE: Ministry of Education
TCM: Traditional Chinese medicine
